# Telomere Length and Risk of Major Adverse Cardiac Events and Cancer in Obstructive Sleep Apnea Patients

**DOI:** 10.3390/cells8050381

**Published:** 2019-04-26

**Authors:** Katarzyna Polonis, Sreeja Sompalli, Christiane Becari, Jiang Xie, Naima Covassin, Phillip J Schulte, Brooke R Druliner, Ruth A Johnson, Krzysztof Narkiewicz, Lisa A Boardman, Prachi Singh, Virend K Somers

**Affiliations:** 1 Department of Cardiovascular Medicine, Mayo Clinic, Rochester, MN 55 905, USA; Polonis.Katarzyna@mayo.edu (K.P.); Sompalli.Sreeja@mayo.edu (S.S.); Ribeiro.Christiane@mayo.edu (C.B.); Xie.Jiang@mayo.edu (J.X.); Covassin.Naima@mayo.edu (N.C.); Singh.Prachi@mayo.edu (P.S.); 2 Department of Hypertension and Diabetology, Faculty of Medicine, Medical University of Gdansk, 80 210 Gdansk, Poland; knark@gumed.edu.pl; 3 Department of Surgery and Anatomy, Ribeirao Preto Medical School, Ribeirão Preto-SP 14049-900, Brazil; 4 Division of Biomedical Statistics and Informatics, Mayo Clinic, MN55 905, USA; Schulte.Phillip@mayo.edu; 5 Division of Gastroenterology and Hepatology, Department of Internal Medicine, Mayo Clinic, Rochester, MN 55 905, USA; Druliner.Brooke@mayo.edu (B.R.D.); Johnson.Ruth4@mayo.edu (R.A.J.); Boardman.Lisa@mayo.edu (L.A.B.)

**Keywords:** obstructive sleep apnea, telomere length, major adverse cardiac events, cancer

## Abstract

Telomere length (TL) is associated with cardiovascular disease (CVD) and cancer. Obstructive sleep apnea (OSA) is also linked to higher risk of CVD and cancer, and to TL. We investigated the association between TL and risk of major adverse cardiac events (MACE) and cancer in OSA patients. We studied 210 individuals undergoing sleep-related studies between 2000 and 2007. Baseline characteristics and follow-up data (available in 164 subjects) were obtained from clinic records. Incidence rates were calculated for the entire group and by OSA status. Hazard ratios were calculated to estimate effects of OSA and TL on risk of MACE and cancer. In total, 32 individuals (20%) developed MACE and/or cancer during 12.7-year follow-up. The OSA group had a higher likelihood of cancer (16.0 vs. 4.9 events per 1000 person-years, *P* = 0.044) but no clear evidence of an elevated incidence of MACE (10.8 vs. 4.8 events per 1000 person-years, *P* = 0.293) compared to the non-OSA group. There was no association between TL and MACE- (HR = 1.01, 95% CI 0.78–1.28), or cancer-risk (HR = 1.18, 95% CI 0.96–1.43). Our study warrants further investigation of any modulating effect of OSA on TL and the risk of MACE and cancer.

## 1. Introduction

Cardiovascular disease (CVD) and cancer are the leading causes of death worldwide, accounting for 31% and 17% of all global deaths, respectively [1]. Obstructive sleep apnea (OSA), a common sleep breathing disorder affecting up to 38% of adults in the general population, is recognized as an independent risk factor for cardiovascular and metabolic disease, including hypertension, cardiac arrhythmias, congestive heart failure, stroke and diabetes [2,3].

Interestingly, there is compelling emerging evidence documenting the association between sleep-disordered breathing, including OSA, and the risk of developing cancer, cancer progression and related mortality [4,5,6]. Recent epidemiological studies have highlighted common risk factors for CVD and cancer, such as obesity and diabetes, suggesting shared pathophysiologic mechanisms [7]. OSA has also been shown to affect telomere length (TL) [8]. These observations highlight potential synergistic links between cancer, cardiovascular diseases and sleep apnea through overlapping molecular mechanisms, such as telomere dynamics.

Telomere shortening is recognized as a fundamental mechanism underlying biological aging and age-related disorders [9]. Telomere length is suggested to accumulate a burden of stressors (such as oxidative stress and inflammation) over the life-time, and therefore better reflect biological age of each individual and disease risk [10,11,12]. Although there are still conflicting findings in the literature, short telomeres are generally proposed to be associated with CVD (arteriosclerosis, coronary artery disease, myocardial infarction etc.) [13,14,15,16,17], while both short and long telomeres were linked with neoplasm onset and progression [18]. Some studies also showed that OSA is associated with altered telomere dynamics, but the direction of the association is contradictory across observational studies. Some of them have linked OSA with telomere shortening [8,19]; however our previous and other studies suggest more complex telomere dynamics, in that telomere length changes may be disease context-dependent [20,21,22,23,24].

Although there is emerging evidence of the value of telomere length in predicting cardiovascular events and cancer, the role of OSA in modifying the link between telomere length and risk of CVD and cancer has not previously been studied. Therefore, in this exploratory hypothesis-generating study, we sought to prospectively investigate the association between telomere length and the risk of major adverse cardiac events (MACE) and cancer in OSA and non-OSA patients. 

## 2. Materials and Methods

### 2.1. Study Population

In this prospective study, we collected data from 210 individuals participating in sleep-related research studies conducted at the Mayo Clinic (Rochester, MN) between 2000–2007. The Mayo Clinic Institutional Review Board approved the study protocol (IRB #15-006260). The enrollment process and telomere findings in this cohort have been described in detail previously [23]. Briefly, all subjects had undergone a polysomnography (PSG) evaluation to confirm or exclude the diagnosis of OSA at the baseline. Baseline information regarding anthropometric and demographic data and medical history was collected from the Mayo medical records and patient questionnaires. Baseline was defined as the date of blood sample collection upon the enrollment to the study.

### 2.2. Clinical Follow-Up and Outcomes

Detailed clinical data related to the follow-up outcomes were abstracted from Mayo Clinic medical records. The primary outcome was a clinical diagnosis of Major Adverse Cardiac Events (MACE) or cancer. MACE was defined as a composite of clinical events including cardiovascular-related death, non-fatal myocardial infarction, congestive heart failure, coronary artery disease (stent thrombosis, target lesion revascularization, target vessel revascularization, coronary artery bypass grafting), and cerebrovascular accident [25]. Cancer was defined as any type of neoplasia. The end of follow up was defined as the date of primary outcome diagnosis or the date of last available clinical record for those who were event-free at the end of observation (November 2018). Patients with no clinical evidence of defined outcomes were censored. Survival time was calculated from the date of baseline to the date of clinical diagnosis of outcome or the date of last clinical record available. Patients with no follow-up information (no medical records available) were excluded from the study. 

### 2.3. Polysomnography Examination

All participants underwent polysomnographic (PSG) evaluation at the Clinical Research and Trials Unit or the Center for Sleep Medicine at the Mayo Clinic in Rochester. Sleep was scored according to standard criteria [26,27]. The apnea-hypopnea index (AHI) was computed as a total number of respiratory events per hour of sleep (n/hr). Individuals with AHI ≥ 5 were classified as OSA group. OSA subjects were further grouped as moderate-to-severe OSA based on AHI ≥ 15 [2].

### 2.4. Leukocyte Telomere Length Measurements

DNA was extracted from peripheral blood samples with the Puregene Blood Kit (Qiagen). Cawthon’s qPCR method was used to determine relative TL [28,29] which was next converted into base pairs (bp) using the validated formula as previously described [23]. We recognize that the calculations derived from telomere qPCR data are used to infer telomere repeat containing products and therefore infer telomere length and do not actually reflect the absolute telomere length.

### 2.5. Statistical Analysis

The distribution of TL was examined, and 3 subjects with TL above 5871 bp (>3 times the interquartile range above the third quartile) were excluded. Data are presented as mean and standard deviation (SD) and percentage. Comparisons between those with and without OSA were assessed using *t*-tests for continuous variables and Pearson chi-square or Fisher’s exact tests for nominal variables. 

Crude MACE and cancer rates per 1000 person-years (incidence density describing the occurrence of a defined outcome) were calculated for the entire sample and by OSA status/categories. A log-rank test compared the survival distribution of OSA and non-OSA subjects. Multivariable Cox proportional hazard regression models were used to estimate hazard ratios (HRs) to describe the association between baseline OSA status and TL on the risk of MACE and the risk of cancer over a long-term follow-up period. Models were adjusted for age, a main determinant of telomere length and a risk factor for both cardiovascular disease and cancer. In the first step, HR was calculated separately for each predictor (OSA status, TL and age); in the second step, adjusted HRs (adjusted for age in a multivariable Model 1 and adjusted for age, BMI, and sex in a multivariable Model 2) were calculated to examine the association between OSA status, TL and the risk of MACE or cancer. No other model adjustments were performed to avoid model overfitting. Median follow up was calculated with the Kaplan–Meier method to estimate median time-to-censoring.

A power calculation was not done a priori as we did not have available data to anticipate an event rate in this sample. However, based on the observed sample size for analysis of 161 subjects, approximate standard deviation for TL of 200 bp, and an event rate of 10% for the endpoint (MACE alone or cancer alone), we would have 80% power to detect an unadjusted hazard ratio of 1.42 per 100 bp for the relationship between TL and the endpoint.

## 3. Results

### 3.1. Population Characteristic

**Figure 1:**
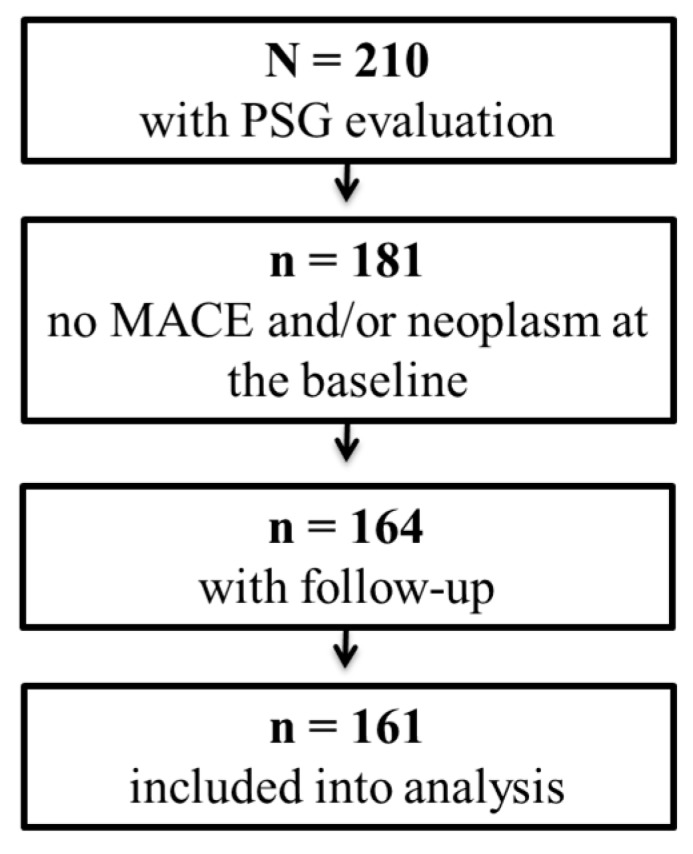
Flow chart of participant inclusion in the study.

Out of 210 subjects included in the study, 181 had no diagnosis of MACE and/or neoplasm at baseline, 164 had available follow-up information (90%), and 3 were identified as extreme outliers. Individuals lost for follow-up were mostly without OSA (n = 14). Finally, 161 patients were included into the analysis (Figure 1). Baseline characteristics of subjects are presented by OSA status in Table 1.

There were significantly more men than women in the OSA group (*P* = 0.002). In the OSA group there were more non-Hispanic Whites than in the non-OSA group (92% vs. 64%, *P* < 0.001). The frequency of post-graduate education among the non-OSA group was more than double the rate in the OSA group (39% vs. 15%, *P* < 0.001). The prevalence of dyslipidemia (*P* = 0.0) and hypertension (*P* = 0.01) was higher in the OSA group. OSA patients were also significantly older (*P* < 0.001), had higher body mass index (*P* < 0.001), higher systolic and diastolic blood pressure (*P* = 0.003 and *P* = 0.030, respectively), and heart rate (*P* = 0.044). The mean AHI for the OSA group was 33.9 ± 27.3 with an average oxygen saturation of 81.8 ± 9.2%. Average oxygen saturation nadir for the moderate-to-severe OSA group was 79.6 ± 9.7%. 

### 3.2. Telomere Length

**Figure 2:**
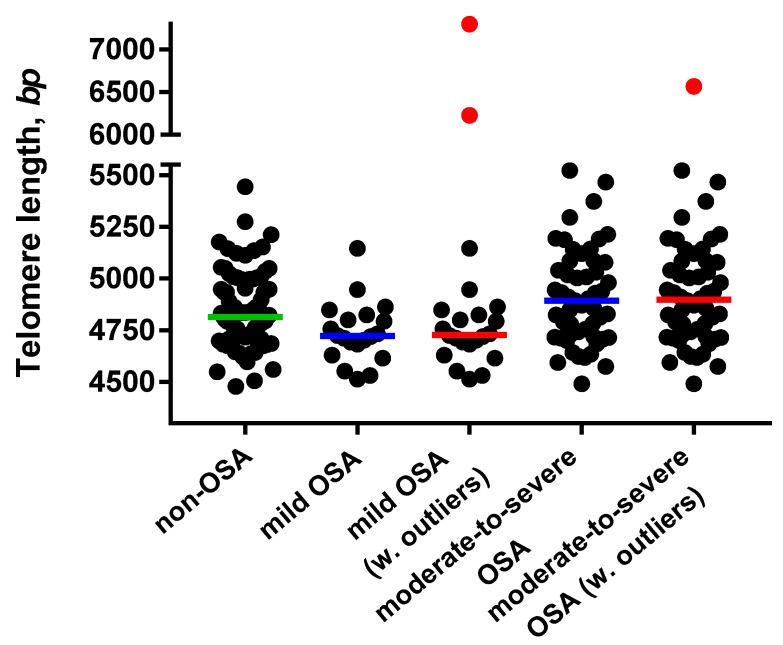
Telomere length dispersion according to obstructive sleep apnea (OSA) severity. Horizontal lines indicate medians: non-OSA (─), mild OSA (─), moderate-to-severe OSA (─). Outliers are marked as (●).

The dispersion of TL according to OSA status is presented in Figure 2. The significance of outliers remains uncertain; however, it is conceivable that TL may be affected by unique factors related to genetic background and environmental exposure not included in this study, as discussed later. 

As previously described in this patient cohort [23], there was no significant difference in TL, on average, between the non-OSA and OSA group (*P* = 0.482); however, a J-shaped association between TL and OSA severity, with the longest average TL in moderate-to-severe OSA (4912 ± 234 bp), and shortest average TL in mild OSA (4739 ± 148 bp) was observed.

### 3.3. MACE and Cancer Risk

During the median follow up period of 12.7 years, 20% of individuals (n = 32) experienced the primary outcome: A new clinical diagnosis of MACE alone (n =14), cancer alone (n = 16), both cancer and MACE (n = 2) (Supplementary Table S1).

**Figure 3:**
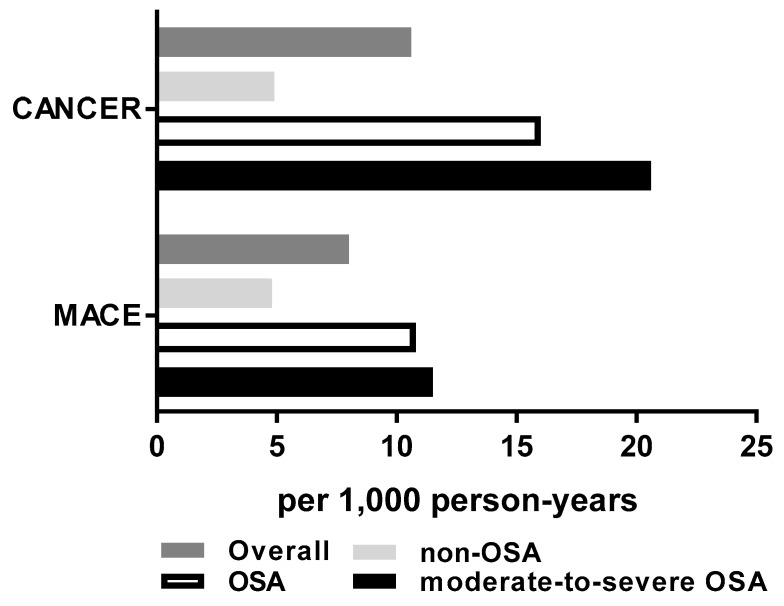
Major adverse cardiac events (MACE) and cancer risk presented as the incidence rates per 1000 person-years. Sample size for non-OSA is n = 88, for OSA is n = 73, and for moderate-to-severe OSA is n = 53.

The overall MACE and cancer risk (incidence density) over the period of follow-up was 8.0 and 10.6 cases per 1000 person-years, respectively. OSA individuals had higher incidence rates of cancer than non-OSA individuals: 16.0 vs. 4.9 events per 1000 person-years (*P* = 0.044) (Figure 3). Rates were numerically higher for the MACE endpoint, but the unadjusted difference was not statistically significant (10.8 vs. 4.8 events per 1000 person-years, *P* = 0.293). Moderate-to-severe OSA status was associated with a 4.2 times higher incidence of cancer compared to the non-OSA individuals (20.6 vs. 4.9 events per 1000 person-years, *P* = 0.006).

Next, Cox proportional hazards regression was used to investigate MACE risk and cancer risk depending on OSA status and TL (Table 2). In an univariate model, age and BMI, but not sex, showed a significant association with risk of cancer and risk of MACE. Each year increase in age was associated with 7% higher risk of MACE (*P* = 0.002) and 8% higher risk of cancer (*P* < 0.001). Each unit increase in BMI was associated with 7% higher risk of MACE (*P* = 0.052) and 5% higher risk of cancer (*P* = 0.032). OSA, regardless of OSA severity, was associated with 3.3 times increased risk of cancer [HR = 3.30, 95% CI (1.08–10.03), *P* = 0.036], but the association was not statistically significant for MACE [HR = 1.87, 95% CI (0.57–6.15), *P* = 0.301].

In an age-adjusted model with TL and OSA status as predictors, TL showed no association with the risk of MACE [HR = 1.01, 95% CI (0.78–1.28), *P* = 0.905], but there was a potentially meaningful association between long TL and cancer risk [HR = 1.18, 95% CI (0.96–1.43, *P* = 0.109]. A point estimate indicated that each 100 bp increase in TL was associated with 18% higher risk of developing cancer. Additional model adjustment for BMI and sex did not change significantly the association between TL and risk of MACE.

The relationship between OSA and risk of cancer [HR = 1.65, 95% CI (0.50–5.41), *P* = 0.409], and risk of MACE [HR = 0.96, 95% CI (0.27–3.48), *P* = 0.954] was not significant in the OSA group overall when controlling for TL and age. Additional adjustment for BMI and sex did not change this observation. A model that further adjusted for smoking status suggested similar relationships for OSA and TL with risk of MACE and risk for cancer. The age-adjusted risk of cancer tended to be higher in individuals with moderate-to-severe OSA than in healthy individuals, but the difference was not statistically significant [HR = 2.04, 95% CI (0.60–6.95), *P* = 0.255].

## 4. Discussion

This study shows that while there was evidence of an association between OSA severity and telomere dynamics in this patient cohort [23], there was no compelling evidence of an association between telomere length and the risk of MACE, but a suggestive, though non-significant, association between telomere length and the risk of cancer in this population.

First, we acknowledge the limited sample size and the relatively small number of events. This resulted in wide confidence intervals of estimates describing the risk of MACE and risk of cancer, indicating a high level of uncertainty of the observed association. Also, because of the lack of complete data, we were not able to account for confounding factors other than age, sex and BMI which may inflate the overall risk of MACE and cancer attributable to other factors such as lifestyle choices, exposure to mutagens and genetic predisposition [30]. These results should thus be interpreted cautiously. However, we anticipate that findings observed in the dataset may represent real effects in OSA populations, and may therefore have implications for the design of clinical and experimental studies aimed to address telomere-related mechanisms underlying cardiovascular and cancer risk in OSA.

Our study adds to growing epidemiological and clinical evidence that patients with sleep breathing disorders, including OSA, tend to have higher rates of cancer [31,32,33]. In this study, we observed that OSA patients, who were free of any neoplasm at baseline, were 3.2 times more often diagnosed with cancer over a median follow-up of 12.7 years. This association agrees with findings from other studies [34,35,36]. Furthermore, we showed that moderate-to-severe OSA was associated with even higher incidence of cancer compared to overall OSA individuals. Our observation is consistent with previous studies which reported that the severity of baseline sleep-disordered breathing is associated with increased cancer incidence and mortality in a dose-response fashion [37,38,39].

The potential link between cancer and OSA is particularly interesting, yet the underlying mechanisms are still poorly understood. Given the established role of hypoxemia in tumorigenesis and cancer progression, the hypothesis that intermittent hypoxemia may be one of the common molecular pathways between both disorders is robust [40]. Animal models of melanoma, breast, and prostate cancer have provided evidence that intermittent hypoxemia, which is a hallmark of OSA, enhances tumorigenesis, tumor growth and metastasis [41,42,43]. This hypothesis is strengthened by epidemiological observations indicating a considerably highly prevalence of OSA in cancer populations. For example, 50% of patients with lung cancer were also diagnosed with moderate-to-severe OSA [44,45]. Some studies have also suggested that sleep disordered breathing may be associated with cancer aggressiveness and worse prognosis [37,46,47].

The cancer promoting effect of OSA has been linked with a number of genetic, molecular and cellular mechanisms promoting a permissive inflammatory microenvironment for tumorigenesis and cancer cell survival by mechanisms potentially related to telomere length [43]. Telomere length defines proliferative potential or the number of cell divisions before crisis leading to cell growth arrest (senescence) or cell death (apoptosis) [48]. It is widely described that short telomeres lead to genomic instability, and therefore an increased risk for cancer. However, there is also growing evidence describing a cancer-telomere length paradox where individuals with long telomeres also showed an increased risk for developing cancer [49]. Whether these individuals also had undiagnosed moderate-to-severe OSA, contributing to both long telomeres and to increased cancer risk, is unknown.

In our study, we detected no statistically significant effect of TL on the risk of MACE; however there was a considerable effect size of TL on the risk of cancer. Furthermore, moderate-to-severe OSA individuals, who had longer telomeres on average, showed higher age-adjusted risk of cancer (but not MACE) than non-OSA individuals. This observation raises the possibility that OSA-related telomere elongation may increase the risk of developing cancer, but moderate the risk of cardiovascular disease [50]. We hypothesize that telomere elongation may be linked to up-regulated telomerase expression and/or activity as an adaptive response to the repetitive oxygenation disturbances related to OSA [51,52].

On the other hand, longer telomeres and a sustained cell proliferation capacity increase the probability of attaining a critical number of genetic mutations [49]. Oxygen desaturations experienced by OSA patients may increase oxidative stress and consequently induce DNA damage and increase risk of genetic mutations leading to genome instability and neoplastic transformation [53]. We speculate that the combination of OSA-mediated telomere elongation and a higher rate of genetic mutation accumulation (related to more severe oxygen desaturations observed in moderate-to-severe OSA) may represent a survival advantage of precancerous cells, and thus predispose OSA patients to higher risk of cancer.

Our study highlights the need for further experimental, clinical, and epidemiological studies to explore these hypotheses further and provide comprehensive evidence of any casual effect of OSA on telomere length and the risk of cancer and cardiovascular events. This may guide the development of screening approaches for early cancer detection in OSA patients.

## Figures and Tables

**Table 1:** Baseline characteristics of the study group.

Variable	Non-OSA n = 88	OSA n = 73	*P-Value*
Telomere length, *bp*	4842 ± 180	4865 ± 226	0.482
Age, *years*	35.6 ± 11.0	47.3 ± 11.9	<0.001
Systolic BP, *mmHg*	124.1 ± 15.3	131.7 ± 15.3	0.003
Diastolic BP, *mmHg*	73.1 ± 9.8	76.4 ± 9.1	0.030
Heart rate, *beats/min*	69.6 ± 11.3	73.9 ± 14.0	0.044
Body mass index, *kg/m^2^*	26.9 ± 5.1	33.0 ± 7.7	<0.001
Male sex, *n (%)*	59 (67%)	64 (87%)	0.002
Diabetes mellitus, *n (%)*	1 (1%)	0 (0%)	1
Dyslipidemia, *n (%)*	6 (7%)	13 (18%)	0.031
Hypertension, *n (%)*	7 (8%)	17 (23%)	0.010
Current smoking, *n (%)*	7 (8%)	5 (7%)	0.775
Race/Ethnicity, *n (%)*			
*African American*	3 (3%)	0 (0%)	0.252
*Asian*	18 (20%)	3 (4%)	0.002
*Pacific Islander*	1 (1%)	0 (0%)	1.0
*White*	56 (64%)	67 (92%)	<0.001
Hispanic/Latino *	7 (8%)	3 (4%)	0.314
*Unknown*	3 (3%)	0 (0%)	0.252
Education, *n (%)*			
*High school graduate*	9 (10%)	17 (23%)	0.025
*Collage graduate/degree*	42 (48%)	44 (60%)	0.112
*Post graduate degree*	34 (39%)	11 (15%)	<0.001
*Unknown*	3 (3%)	1 (1%)	0.627
Apnea-hypopnea index, *n/h*	1.0 ± 1.3	33.9 ± 27.3	<0.001
Minimum blood O_2_ saturation, *%*	90.1 ± 5.2	81.8 ± 9.2	<0.001

OSA-obstructive sleep apnea, *bp*–base pair, BP–blood pressure, * Hispanic/Latino includes subjects of any race that identify as Hispanic/Latino ethnicity.

**Table 2:** Hazard Ratios (HRs) for the development of MACE and cancer with OSA status, age and TL as individual (univariable model) and combined predictors (multivariable).

	Univariable Model		Multivariable Model 1		Multivariable Model 2	
MACE	HR (95% CI)	*P-Value*	HR (95% CI)	*P-Value*	HR (95% CI)	*P-Value*
OSA vs. non-OSA	1.87 (0.57–6.15)	0.301	0.96 (0.27–3.48)	0.954	0.51 (0.12–2.19)	0.368
TL, *per 100 bp*	1.02 (0.79–1.29)	0.877	1.01 (0.78–1.28)	0.905	1.05 (0.81–1.34)	0.705
Age, *yrs*	1.07 (1.03–1.12)	0.002	1.07 (1.02–1.12)	0.003	1.07 (1.02–1.13)	0.003
BMI, *kg/m^2^*	1.07 (0.99–1.13)	0.052			1.08 (1.00–1.15)	0.041
Male sex	2.75 (0.36–21.20)	0.333			3.92 (0.47–32.64)	0.205
**CANCER**						
OSA vs. non-OSA	3.30 (1.08–10.03)	0.036	1.65 (0.50–5.41)	0.409	1.73 (0.44–6.72)	0.428
TL, *per 100 bp*	1.15 (0.93–1.39)	0.175	1.18 (0.96–1.43)	0.109	1.21 (0.98–1.48)	0.068
Age, *yrs*	1.08 (1.04–1.12)	<0.001	1.07 (1.03–1.12)	<0.001	1.07 (1.03–1.12)	<0.001
BMI, *kg/m^2^*	1.05 (1.00–1.10)	0.032			1.03 (0.96–1.09)	0.422
Male sex	0.58 (0.21–1.63)	0.301			0.45 (0.13–1.54)	0.204

MACE—major adverse cardiac events, OSA—obstructive sleep apnea, TL—telomere length, *bp*—base pair, BMI—body mass index.

## Supplementary Materials

The following are available online at https://www.mdpi.com/2073-4409/8/5/381/s1, Table S1: List of neoplasms in the dataset.
