# Supplementary material for: Telomere Length and Risk of Major Adverse Cardiac Events and Cancer in Obstructive Sleep Apnea Patients
Source: Cells. 2019 Apr 26;8(5):381. doi: 10.3390/cells8050381 (PMC6562838; doi:10.3390/cells8050381)
Supplement: Supplementary file 1 [file cells-08-00381-s001.pdf]

Supplementary Table S1 List of neoplasms in the dataset

Neoplasm type	N
chronic lymphocytic leukemia	1
malignant neoplasm of prostate	3
malignant neoplasm of breast	2
malignant carcinoid tumors of bronchus and lung	1
malignant neoplasm of corpus uteri	1
malignant neoplasm of skin	
melanoma	1
basal cell carcinoma	3
squamous cell carcinoma with basal cell carcinoma	1
not specified type	5
